# Supplementary material for: Malignancy Prediction Capacity and Possible Prediction Model of Circulating Tumor Cells for Suspicious Pulmonary Lesions
Source: J Pers Med. 2021 May 21;11(6):444. doi: 10.3390/jpm11060444 (PMC8223995; doi:10.3390/jpm11060444)
Supplement: Supplementary file 1 [file jpm-11-00444-s001.zip › jpm-1199198-supplementary.pdf]

Supplement Figure S1. Study protocol and inclusion-exclusion criteria.

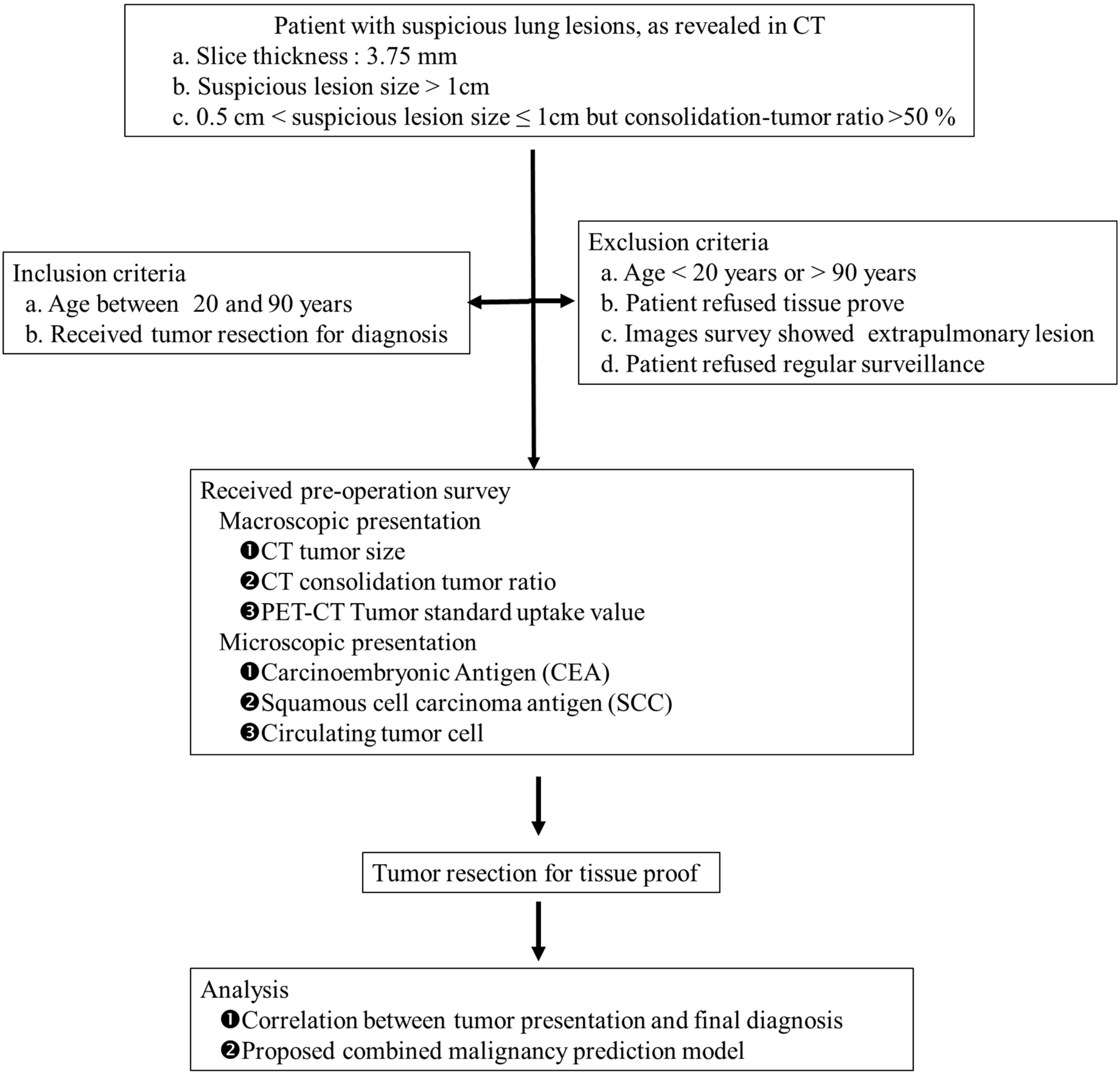

Supplement Table S1. Logistic regression for model selection

Logistic regression for all tumor presentations, except tumor size and CTC are listed below. None of them has statistical significance for malignancy prediction.

Analysis of Maximal Likelihood Estimates				
Parameter	Estimates	Standard errors	95 % confidence interval	P value (Chi-Square)
Log (Odds ratio for C/T ratio > 50%)	-24.9674	0.0000	(-24.9674,-24.9674)	0.9525
Odds ratio for C/T ratio > 50%	NA	NA	NA	
Log (Odds ratio for tumor SUV > 2.5)	0.5108	1.2824	(-2.0026,3.0242)	0.6904
Odds ratio for tumor SUV >2.5	1.6667	2.1373	(0.1350,20.5776)	
Log (Odds ratio for CEA > 3.4)	25.1681	276889.0	(-542667,542717.7)	0.9999
Odds ratio for CEA > 3.4	NA	NA	NA	
Log (Odds ratio for SCC >3.5)	NA	NA	NA	NA
Odds ratio for SCC >3.5	NA	NA	NA	
Log (Odds ratio for N/L ratio > 60%)	-0.9008	1.0527	(-2.9640,1.1625)	0.3922
Odds ratio for N/L ratio > 60 %	0.4063	0.4277	(0.0516,3.1978)	
Log (Odds ratio for tumor size ≥0.7)	2.7081	1.5348	(-0.3001, 5.7162)	0.0777
Odds ratio for tumor size ≥0.7	15.0000	23.0217	(0.7408, 303.7386)	
Log (Odds ratio for CTC ≥3)	2.5123	1.2130	(0.1348,4.8898)	0.0384
Odds ratio for CTC ≥3	12.3333	14.9609	(1.1443,132.9303)	

Supplement Table S2. Detail of receiver operating characteristic curve for tumor size.
Cutoff value of tumor size was chosen 0.7 cm because of highest sensitivity.

Obs	_PROB_	_POS_	_NEG_	_FALPOS_	_FALNEG_	_SENSIT_	_1MSPEC_	_SPECIF_	LOGIT	CUT_POINT	Y
1	0.90330	36	2	2	10	0.78261	0.50	0.50	2.23446	1.30014	0.28261
2	0.90557	35	2	2	11	0.76087	0.50	0.50	2.26070	1.40014	0.26087
3	0.88865	45	1	3	1	0.97826	0.75	0.25	2.07702	0.70014	0.22826
4	0.89122	44	1	3	2	0.95652	0.75	0.25	2.10326	0.80014	0.20652
5	0.89374	43	1	3	3	0.93478	0.75	0.25	2.12950	0.90014	0.18478
6	0.94993	8	4	0	38	0.17391	0.00	1.00	2.94293	4.00011	0.17391
7	0.90779	31	2	2	15	0.67391	0.50	0.50	2.28694	1.50014	0.17391
8	0.90996	30	2	2	16	0.65217	0.50	0.50	2.31318	1.60014	0.15217
9	0.92753	18	3	1	28	0.39130	0.25	0.75	2.54933	2.50013	0.14130
10	0.92927	17	3	1	29	0.36957	0.25	0.75	2.57557	2.60013	0.11957
11	0.89862	40	1	3	6	0.86957	0.75	0.25	2.18198	1.10014	0.11957
12	0.95237	5	4	0	41	0.10870	0.00	1.00	2.99541	4.20011	0.10870
13	0.93098	16	3	1	30	0.34783	0.25	0.75	2.60181	2.70013	0.09783
14	0.90098	39	1	3	7	0.84783	0.75	0.25	2.20822	1.20014	0.09783
15	0.95354	4	4	0	42	0.08696	0.00	1.00	3.02165	4.30011	0.08696
16	0.91209	27	2	2	19	0.58696	0.50	0.50	2.33942	1.70013	0.08696
17	0.93265	15	3	1	31	0.32609	0.25	0.75	2.62805	2.80012	0.07609
18	0.95798	3	4	0	43	0.06522	0.00	1.00	3.12661	4.70011	0.06522
19	0.93743	13	3	1	33	0.28261	0.25	0.75	2.70677	3.10012	0.03261
20	0.96103	1	4	0	45	0.02174	0.00	1.00	3.20533	5.00011	0.02174
21	0.93895	12	3	1	34	0.26087	0.25	0.75	2.73301	3.20012	0.01087
22	0.91417	23	2	2	23	0.50000	0.50	0.50	2.36566	1.80013	0.00000
23	0.88603	46	0	4	0	1.00000	1.00	0.00	2.05078	0.60014	0.00000
24	0.94043	11	3	1	35	0.23913	0.25	0.75	2.75925	3.30012	-0.01087
25	0.91621	22	2	2	24	0.47826	0.50	0.50	2.39189	1.90013	-0.02174
26	0.94331	10	3	1	36	0.21739	0.25	0.75	2.81173	3.50012	-0.03261
27	0.91820	21	2	2	25	0.45652	0.50	0.50	2.41813	2.00013	-0.04348
28	0.92015	20	2	2	26	0.43478	0.50	0.50	2.44437	2.10013	-0.06522
29	0.92574	19	2	2	27	0.41304	0.50	0.50	2.52309	2.40013	-0.08696

Supplement Table S3. Detail of receiver operating characteristic curve for CTC.

Cutoff value of tumor size was chosen 3 cells/ ml because of high sensitivity and specificity

Obs	_PROB_	_POS_	_NEG_	_FALPOS_	_FALNEG_	_SENSIT_	_1MSPEC_	_SPECIF_	LOGIT	CUT_POINT	Y
1	0.95474	28	4	0	18	0.60870	0.00	1.00	3.0490	6.7004	0.60870
2	0.95831	27	4	0	19	0.58696	0.00	1.00	3.1349	7.0005	0.58696
3	0.87967	37	3	1	9	0.80435	0.25	0.75	1.9893	3.0002	0.55435
4	0.96367	25	4	0	21	0.54348	0.00	1.00	3.2781	7.5005	0.54348
5	0.96836	23	4	0	23	0.50000	0.00	1.00	3.4213	8.0005	0.50000
6	0.97918	21	4	0	25	0.45652	0.00	1.00	3.8510	9.5006	0.45652
7	0.89402	32	3	1	14	0.69565	0.25	0.75	2.1325	3.5003	0.44565
8	0.90684	31	3	1	15	0.67391	0.25	0.75	2.2757	4.0003	0.42391
9	0.98191	19	4	0	27	0.41304	0.00	1.00	3.9942	10.0006	0.41304
10	0.92838	30	3	1	16	0.65217	0.25	0.75	2.5621	5.0003	0.40217
11	0.98635	17	4	0	29	0.36957	0.00	1.00	4.2806	11.0007	0.36957
12	0.80478	40	2	2	6	0.86957	0.50	0.50	1.4164	1.0001	0.36957
13	0.94524	28	3	1	18	0.60870	0.25	0.75	2.8485	6.0004	0.35870
14	0.84591	39	2	2	7	0.84783	0.50	0.50	1.7029	2.0002	0.34783
15	0.99785	14	4	0	32	0.30435	0.00	1.00	6.1423	17.5010	0.30435
16	0.99814	13	4	0	33	0.28261	0.00	1.00	6.2855	18.0010	0.28261
17	0.99886	12	4	0	34	0.26087	0.00	1.00	6.7724	19.7011	0.26087
18	0.99941	11	4	0	35	0.23913	0.00	1.00	7.4312	22.0012	0.23913
19	0.99956	10	4	0	36	0.21739	0.00	1.00	7.7176	23.0013	0.21739
20	0.99978	8	4	0	38	0.17391	0.00	1.00	8.4336	25.5014	0.17391
21	0.99981	7	4	0	39	0.15217	0.00	1.00	8.5768	26.0014	0.15217
22	0.99989	6	4	0	40	0.13043	0.00	1.00	9.1496	28.0015	0.13043
23	0.99997	5	4	0	41	0.10870	0.00	1.00	10.2953	32.0018	0.10870
24	1.00000	4	4	0	42	0.08696	0.00	1.00	12.7298	40.5022	0.08696
25	1.00000	3	4	0	43	0.06522	0.00	1.00	13.0162	41.5022	0.06522
26	1.00000	2	4	0	44	0.04348	0.00	1.00	14.3051	46.0025	0.04348
27	1.00000	1	4	0	45	0.02174	0.00	1.00	22.5252	74.7040	0.02174
28	0.75584	46	0	4	0	1.00000	1.00	0.00	1.1300	0.0001	0.00000
